# Scanning probe microscopy studies on the adsorption of selected molecular dyes on titania

**DOI:** 10.3762/bjnano.7.156

**Published:** 2016-11-09

**Authors:** Jakub S Prauzner-Bechcicki, Lukasz Zajac, Piotr Olszowski, Res Jöhr, Antoine Hinaut, Thilo Glatzel, Bartosz Such, Ernst Meyer, Marek Szymonski

**Affiliations:** 1 Research Centre for Nanometer-scale Science and Advanced Materials (NANOSAM), Institute of Physics, Faculty of Physics, Astronomy and Applied Computer Science, Jagiellonian University, Łojasiewicza 11, 30-348 Krakow, Poland; 2 Department of Physics, University of Basel, Klingelbergstr. 82, 4056 Basel, Switzerland

**Keywords:** dye molecules, perylene-3,4,9,10-tetracarboxylic dianhydride (PTCDA), phtalocyanines, porphyrins, rutile, scanning probe microscopy, scanning tunneling microscopy (STM), titanium dioxide (TiO_2_)

## Abstract

Titanium dioxide, or titania, sensitized with organic dyes is a very attractive platform for photovoltaic applications. In this context, the knowledge of properties of the titania–sensitizer junction is essential for designing efficient devices. Consequently, studies on the adsorption of organic dyes on titania surfaces and on the influence of the adsorption geometry on the energy level alignment between the substrate and an organic adsorbate are necessary. The method of choice for investigating the local environment of a single dye molecule is high-resolution scanning probe microscopy. Microscopic results combined with the outcome of common spectroscopic methods provide a better understanding of the mechanism taking place at the titania–sensitizer interface. In the following paper, we review the recent scanning probe microscopic research of a certain group of molecular assemblies on rutile titania surfaces as it pertains to dye-sensitized solar cell applications. We focus on experiments on adsorption of three types of prototypical dye molecules, i.e., perylene-3,4,9,10-tetracarboxylic dianhydride (PTCDA), phtalocyanines and porphyrins. Two interesting heteromolecular systems comprising molecules that are aligned with the given review are discussed as well.

## Introduction

Today it comes as no surprise that photovoltaic devices can be made of materials other than silicon. Nanocrystalline materials accompanied by organic molecules or conducting polymers offer several advantages, e.g., they are relatively cheap to fabricate and can be used on flexible substrates [1]. The use of organic sensitizers allows even wide-band-gap semiconductors to be used in photovoltaic applications. Semiconductors with large band gaps offer stability against photocorrosion at the expense of decreased sensitivity to the visible spectrum. A good example of this type of material is titanium dioxide, which has a band gap of 3.0–3.2 eV and absorbs only the ultraviolet part of the solar spectrum. Thus, bare TiO_2_ used in photovoltaic applications has low conversion efficiencies [1]. However, when the surface of a wide-band-gap material is covered with a sensitizer that absorbs light in the visible spectrum and enables charge transfer through the semiconductor–adsorbate interface, the situation changes dramatically. The optical absorption, and charge separation and transport functions are separated. Now, the properties of the semiconductor–sensitizer junction may increase the conversion efficiency in the photovoltaic device. In view of this, it is essential to study the adsorption properties and the charge transfer of organic dyes on the surfaces of wide-band-gap materials.

First, one may try to search for the factors responsible for the formation of ordered monolayers. These factors could be of different origins and depend on the following: (1) the preparation procedure (e.g., the temperature of a substrate, the specific rate of deposition, or the necessity of some surface pre-treatment), (2) the structure of the deposited molecule (e.g., specific anchoring groups), and (3) a particular balance of the molecule–molecule and molecule–substrate interactions (e.g., different faces of the same bulk material may have distinct properties, or at a high coverage limit, molecule–molecule interactions may lead to a reorientation of the molecules). Obviously, it is naïve to claim that a single factor is responsible for the arrangement of molecules in an ordered layer. The form of the final pattern depends on all of the mentioned determinants, but some of them still play a pivotal role.

Second, once the molecular layers are obtained, one may try to seek for correlations between the morphology of the layer and the electrical properties of the junction. An interesting issue is the evolution of excited charge carriers in the molecular assemblies induced by incoming light. Here, the charge transfer through the semiconductor–molecule junction is crucially important. Additionally, it is important to determine the feasibility of post-processing the molecular layer, as well as its stability against high temperatures and the ambient environment.

Needless to say, there are many wide-band-gap materials that are studied in the context of photovoltaic applications. However, among them, titanium dioxide seems to be favoured. Indeed, since O’Regan and Grätzel published their seminal paper [2], the interest surrounding dye-sensitized solar cells (DSSC) utilizing titania as a semiconducting electrode has consistently increased [3–12]. In the following paper, we review some of the recent research of molecular assemblies on rutile titania surfaces as it pertains to dye-sensitized solar cell applications.

## Review

### Prototypical systems

**Figure 1:**
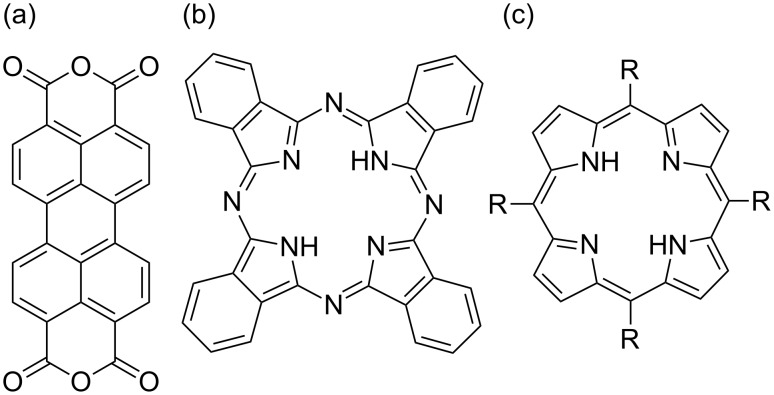
The discussed molecular species (a) PTCDA, (b) metal-free phthalocyanine (H_2_Pc), in which the central hydrogen atoms may be substituted by a metal atom, e.g., Cu, Co, Fe, giving rise to CuPc, CoPc, FePc, respectively, (c) porphyrin, in which the central hydrogen atoms may be substituted by a metal atom giving rise to metal porphyrins; R stands for different ligands.

In any discipline, experience is gained through studying prototypical systems. Among the organic dyes used for sensitization applications, there are many that are considered prototypical. Here we review experiments on three types of molecules: perylene-3,4,9,10-tetracarboxylic dianhydride (PTCDA), phthalocyanines (Pcs) and porphyrins (Figure 1). All of these molecules are dyes absorbing in the visible range, and all of them are used in photovoltaic applications [13–21]. They are also intensively studied using various surface sensitive methods. Surprisingly, however, for each type of molecule mentioned, most reports refer to their adsorption on metallic substrates (for example, see PTCDA [22] and the references therein, or Pcs and porphyrins [23–24] and the references therein). In the context of photovoltaic applications, studies on the adsorption of organic dyes on titania surfaces are necessary.

In this paper, we very briefly describe each of the reviewed molecular species without discussing their properties in detail. More elaborate descriptions may be found elsewhere (for PTCDA see [22,25–26] and for Pcs and porphyrins, see [23]).

The PTCDA molecule is a derivative of perylene and therefore consists of a perylene core and two anhydride groups at each end (see Figure 1a). The perylene core is made up of five benzene rings fused into a planar structure. An anhydride group consists of two carbon atoms interlinked through an oxygen atom, and two additional oxygen atoms, each coupled by a double bond to a single carbon atom.

Phthalocyanines are cyclic aromatic molecules (see Figure 1b). They comprise four isoindole groups coupled through nitrogen atoms. An isoindole group consists of a benzene ring attached to a pyrrole ring. The central void can be occupied by a metal atom, e.g., Cu, Co, and Fe, giving rise to CuPc, CoPc, and FePc molecules, respectively. It is possible that, instead of a metal atom in the void, two hydrogen atoms may exist bonded to the nitrogen of the pyrrole group; this is referred to as a metal-free phthalocyanine (H_2_Pc).

Porphyrins are cyclic aromatic molecules. They are derivatives from porphin, which comprises four pyrrole groups coupled through methine bridges, and substituted porphins are called porphyrins (see Figure 1c). Furthermore, hydrogen atoms in the central void may be substituted by a metal atom, giving rise to metal complexes.

### PTCDA molecules

**Figure 2:**
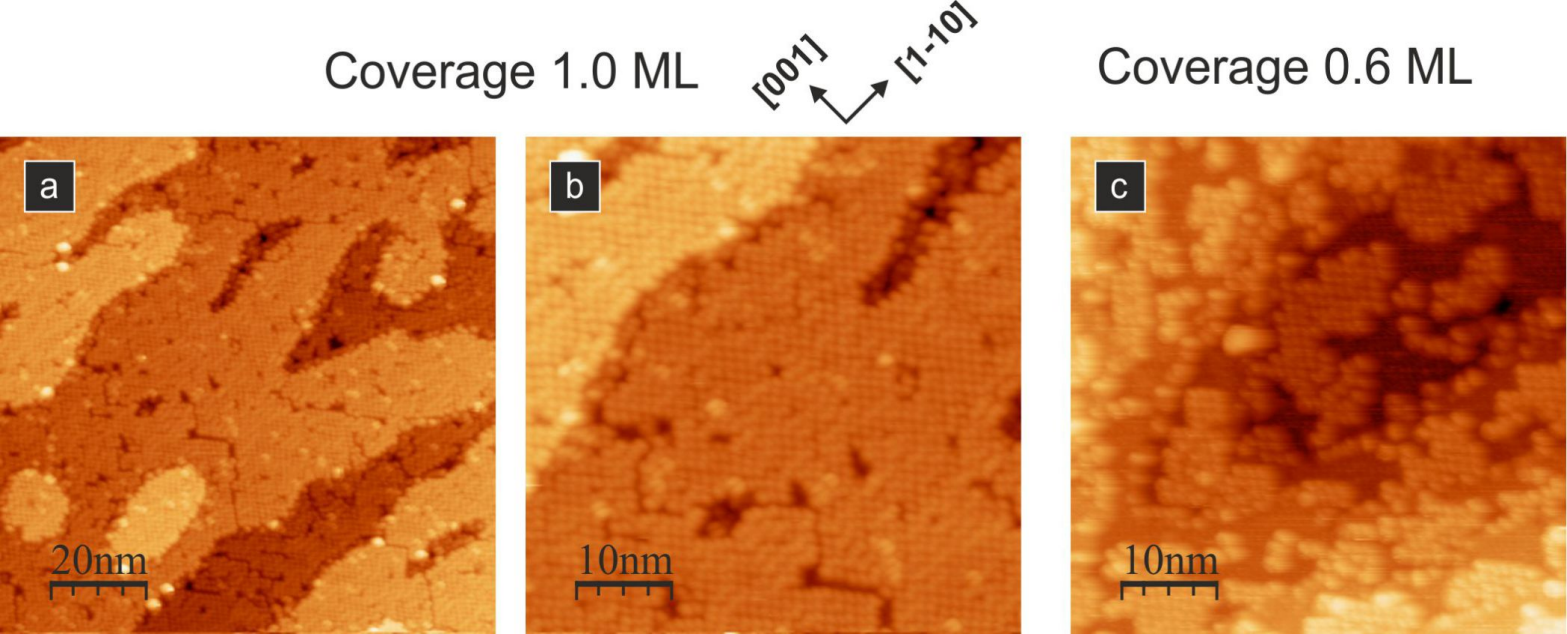
Empty-state STM images of the densely packed molecular structures. (a) and (b): the closed layer PTCDA structure obtained when the molecules are adsorbed on a sample kept at elevated to 100 °C temperature; (c) 0.6 ML of PTCDA molecules adsorbed on the sample kept at 100 °C. All STM images were acquired with a 2 pA tunnelling current and a 2.0 V bias voltage. The figure has been adapted from [31].

Several experimental reports have been devoted to studying PTCDA assemblies on TiO_2_ surfaces [27–33]. Komolov et al. [27] and Cao et al. [29–30] investigated very high coverage densities, i.e., multilayer assemblies (up to a few nanometres thick) of molecules on the (110) face of rutile titania using spectroscopic techniques. For a few nanometre thick overlayers, the formation of an interfacial potential barrier due to band bending in the substrate, the molecular polarization in the organic film, and an increase in the work function have been reported [27]. More detailed analyses of the electronic structure, molecular orientations, energy level alignment and charge transfer dynamics have been provided by Cao et al. [29–30]. The authors showed that strong coupling between the PTCDA molecules and the TiO_2_ substrate results in charge transfer time scales of the order of 8–20 fs [30]. Additionally, ordering of the molecules in the layers is shown to vary from a slightly tilted geometry, to a disordered one, and to a nearly flat-lying geometry as the coverage density increases from the submonolayer to monolayer, and to multilayer regimes [29]. These results can be confronted with scanning tunnelling microscopy studies discussing the influence of dispersion forces on the supramolecular ordering of the molecules in a layer described by Godlewski et al. [28]. These authors have shown that at a low coverage, below 0.7 ML, molecules form poorly ordered layers of physisorbed species. Interestingly, an increased density of the molecular adsorbates leads to changes in the adsorption mode from physisorption to chemisorption, leaving the molecules arranged in a well-ordered brick-wall-like structure. In a more recent study, the same authors examined the influence of the substrate temperature on the ordering in the formed molecular layers (see Figure 2), showing that it is possible to obtain homogeneous molecular islands by annealing PTCDA/TiO_2_(110) at 100 °C [31]. There are also reports concerning the adsorption of PTCDA molecules on the (011) face of rutile [32–33]. For submonolayer coverage, a very narrow range of substrate temperatures has yielded the formation of molecular chains [32]. At higher densities, large disordered areas in the first layer were observed and the formation of the second well-ordered layer was uncertain [33].

It is worth to mention quite recent results reported for another perylene-derivative, namely perylene di-imide (PTCDI) [34–35]. The difference in the molecular structure between PTCDA and PTCDI seems to be small, i.e., an oxygen atom linking carbon atoms in an anhydride group is exchanged by pyrrolic (N–H) nitrogen. However, the local molecular orientation of PTCDI molecules on the TiO_2_(110) surface only partially resembles that observed for PTCDA molecules [28,35]. The long molecular axis in case of both PTCDI and PTCDA is parallel to the [001] direction. Yet, the molecular plane of PTCDI is tilted by an angle of ca. 35° off the surface, in contrast to flat-lying PTCDA. That tilting allows for denser accommodation of molecules on the surface in comparison to what was reported for PTCDA. In such an arrangement molecules from neighbouring rows adapt a closely π-stacked geometry. Furthermore, PTCDI molecules interact strongly with the substrate and eventually form domains that have a (1 × 5) molecular superstructure [34]. Interestingly, it appeared possible to study charge donation from the substrate to the molecular adsorbates in the PTCDI/TiO_2_(110) system. Excess electrons in rutile TiO_2_(110) are introduced either by the formation of oxygen vacancies [36], which are point defects commonly found in TiO_2_(110) surfaces, or by doping [37], and are redistributed among multiple Ti lattice sites in the subsurface layers [36–41]. Thus, a defect state in the band gap is formed. Such delocalized charges may be extracted from the surface and lead to reduction of molecular adsorbates. Lanzilotto et al. [35] have shown that the interaction range of the excess electrons is limited to a single unit cell, i.e., ca. 1 nm^2^, and, hence, reduced PTCDI is identified as single molecules within the (1 × 5) islands.

### Pc molecules

There are very few reports available for TiO_2_ and phthalocyanines in planar heterojunction solar cell applications (see [42] and the references therein). In a few cases among these studies, scanning probe microscopy was used to examine the properties of the Pc/TiO_2_ interface itself [33,43–47] and to investigate the heteromolecular systems in which phthalocyanine molecules formed a second layer [48–49]. Copper phthalocyanine (CuPc) appears to be the most extensively studied Pc derivative on titania surfaces [27,33,43,46]. Other accounts refer to CoPc/TiO_2_(110)[44], FePc/TiO_2_(110) [45], and metal-free Pc/TiO_2_(110) [47] systems.

**Figure 3:**
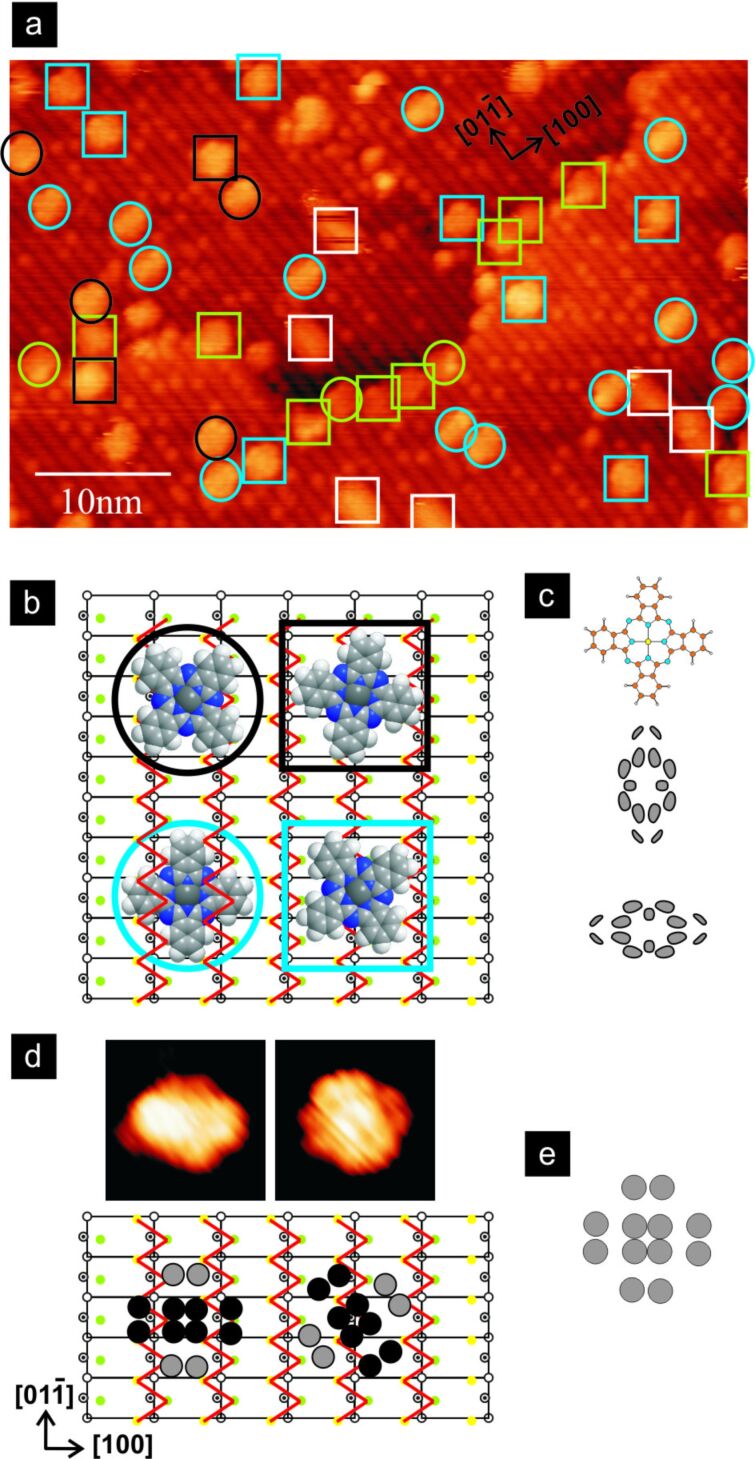
The adsorption geometries of CuPc molecules on the TiO_2_(011) substrate (coverage: 0.06 ML): (a) a submolecularly resolved empty-state STM image, (the squares are molecules adsorbed on oxygen zigzag rows, and the circles are molecules adsorbed in between the zigzag rows), (b) a schematic illustration of stable adsorption geometries on terraces, (c) a schematic image of a CuPc molecule and two orbitals forming the LUMO, (d) STM images of molecules exhibiting two-fold symmetry and their schematic illustration, (e) a schematic illustration of the STM appearance of a CuPc molecule. All scans: bias voltage 2.0 V, tunnelling current 2 pA. Colour coding: white – mobile molecules, green – molecules located at the step edges, black and blue – different azimuthal angle of the molecules. The figure has been adapted with permission from [33], copyright 2011 AIP Publishing LLC.

Let us first take a look at the results published for a CuPc/TiO_2_ system. For CuPc overlayers that were a few nanometres thick on TiO_2_(110), a decrease in the work function values was reported [27]. Wang, Ye and Wu studied the adsorption of CuPc molecules on cross-linked (110)-(1 × 2) and (210) rutile titania surfaces [46]. At a low coverage, the molecules sparsely lay flat at the link sites and lay tilted in the troughs between the [001] rows on a cross-linked (110)-(1 × 2) surface; whereas, on a (210) surface they are tilted in the defect-free areas and lay flat at the defect sites. For regions free of defects on the (210) surface, molecules are preferentially adsorbed at the step edges. In defect areas, the surface defects compete with the step edges to adsorb molecules. It seems that in each of these adsorption geometries, the molecule–substrate interactions are mediated by the π-orbitals of a molecule extending in the direction perpendicular to the molecular plane. At higher coverage, 2D self-assembled structures were reported for both surfaces [46]. Additionally, adsorption on the (011) face at different coverage densities has been investigated [33,43]. At a submonolayer coverage, molecules are predominantly found at the step edges and occasionally at the surface defects [33]. Only the molecules adsorbed on step edges running along surface rows have tilted geometries; the other molecules adopt a flat-lying configuration. At low coverage densities, CuPcs are very mobile; nevertheless, it was possible to analyse the adsorption configurations of flat-lying molecules in detail. Approximately 55% of all of the molecules adsorb with their Cu atom located over the oxygen surface rows. The remaining 45% of the molecules have their copper atom located in between the surface rows. Additionally, the molecules may be oriented differently with respect to the surface rows, leading to further divisions of these two major groups that end in a large variety of possible configurations (Figure 3). Again, it seems plausible to assume that, in adopting these configurations, the interactions mediated by the π-orbitals of the molecule extending in the direction perpendicular to the molecular plane plays a pivotal role. At monolayer coverage, the molecules form a quasi-ordered wetting layer that includes regions comprising parallel molecular lines and regions having chessboard-like structures [33]. The further increase in the amount of deposited organic material leads to the formation of the second layer [33,43]. When molecules are evaporated on the substrate kept at room temperature, the CuPcs in the second layer arrange in two coexisting phases with the majority of molecules lying with their plane parallel to the substrate surface. Post-deposition annealing at 200 °C leads to a reorientation of the molecules in the second layer into an upright geometry [43].

When deposited on a TiO_2_(110) surface, phthalocyanine molecules with cobalt atom substituents adsorb most probably in a flat-lying geometry [44], which aligns with the previously discussed case of CuPcs. Similarly, to the CuPcs on the TiO_2_(011) surface, CoPcs on the (110) face can be classified into two major groups, i.e., those of molecules with Co atoms centred on top of the Ti rows and those centred on the oxygen rows. Furthermore, under specific conditions, CoPc molecules are immobilized on the surface without aggregation, i.e., when deposited with a low rate on a substrate kept at an elevated temperature [44]. The authors ascribe the immobilization of the molecules to the formation of a chemical bond via the dehydrogenation of the eight H atoms of the isoindole ligands, as a result of the increased substrate temperature during the deposition. Next, Ishida and Fujita investigated the electronic properties of individual molecules. The immobilized molecules locally introduced gap states and significantly reduced the surface band gap [44]. At higher coverage densities, CoPcs formed a second layer in which the presence of π-stacked aggregates was suggested. The formation of the first layer is dominated by molecule–substrate interactions; whereas, in the formation of the second layer, molecule–molecule interactions play a key role.

A flat-lying geometry seems to be a common feature for the Pc molecules in the first layer adsorbed on the TiO_2_ surfaces. It is also the case for FePcs deposited onto TiO_2_(110), as reported by Palmgren and co-workers [45]. These authors, in addition to investigating the growth mode of the FePc overlayers, also analysed their electronic structure in detail. The molecular films grow in a layer-plus-island mode, and in the observed layers, the molecular plane is parallel to the surface [45]. The molecules in the first layer are strongly coupled, whereas the FePcs in the second layer are not severely affected by bonding to the surface and exhibit bulk-like electronic properties. The electronic properties of the second and subsequent layers are favourable for DSSC applications. However, the strong coupling of the first layer, resulting in the alteration of the electronic structure and charge transfer from the molecules, is a significant disadvantage [45].

The results obtained for metal-free phthalocyanines are in line with those mentioned above. Molecules in the first layer adsorb in a flat-lying geometry and are aligned with the rows of the bridging oxygen parallel to the [001] direction on the (110) face [47]. The formation of a well-ordered layer was not reported. Palmgren et al. [47] discussed in detail the strong bonds formed between the molecules in the first layer and the substrate, which are induced through a post-deposition annealing step. As-deposited molecules are extremely mobile and rather weakly interact with the substrate. Thermal treatment of the sample leads to the detachment of hydrogen atoms in the central void of the H_2_PC molecules. That in turn results in the chemisorption and immobilization of molecules on the surface. Based on results from high-resolution photoelectron spectroscopy measurements, the authors concluded that the interaction between the molecules in the first monolayer and the substrate comes from the π-electrons of isoindole group and the bridging oxygen atoms on the surface. The bonding between the first and second monolayers is of a weaker van der Waals type. Strong interactions of the molecules in the first layer with the substrate are unfavourable in possible solar cell applications [47]. However, the molecules in the second layer seem to be decoupled from the substrate. The single molecular layer is a good enough buffer for the subsequent layers [50].

Interestingly, there is a single article about ZnPc molecules adsorbed on TiO_2_(110) surface reporting a tilted adsorption geometry in the first layer of molecules [51]. Yu et al. used the photoelectron spectroscopy (PES) and the near-edge X-ray absorption spectroscopy (NEXAFS) to investigate the ZnPc/TiO_2_ system. They conclude that molecules in the first layer bind with the molecular plane 30° from the surface and the tilt angle increases to 33° at higher coverage [51]. However, already the authors suggest further studies on molecular adsorption in the first layer as molecules may be distorted by some specific molecule–substrate interactions. Scanning probe experiments on ZnPc/TiO_2_ would be advantageous in this regard. In agreement with previous studies on Pc/TiO_2_ discussed above, it is found that the ZnPc molecules in the interface layer interact strongly with the substrate, i.e., charge is transferred from the molecule to the substrate. As a consequence, the molecules in the first layer differ much from molecules in the higher overlayers in terms of electronic structure [51].

### Porphyrin molecules

Porphyrin molecules have been intensively studied on metal substrates (for a recent review, see [23–24]). However, studies devoted to understanding the interface properties of an organic layer on titania surfaces are not so numerous [49,52–60]. Among these studies, three reports outline the adsorption properties and behaviour of metal-free porphyrins on TiO_2_(110) surfaces [52–54], five study various zinc porphyrins on both the (110) and (011) surfaces [55–59], and just a single report covers copper porphyrins deposited on the (110) face of the rutile [60]. Recently, zinc porphyrins have also been used in a heteromolecular system as underlayers for the adsorption of CuPc on TiO_2_. We will discuss that system in detail in the next section.

**Figure 4:**
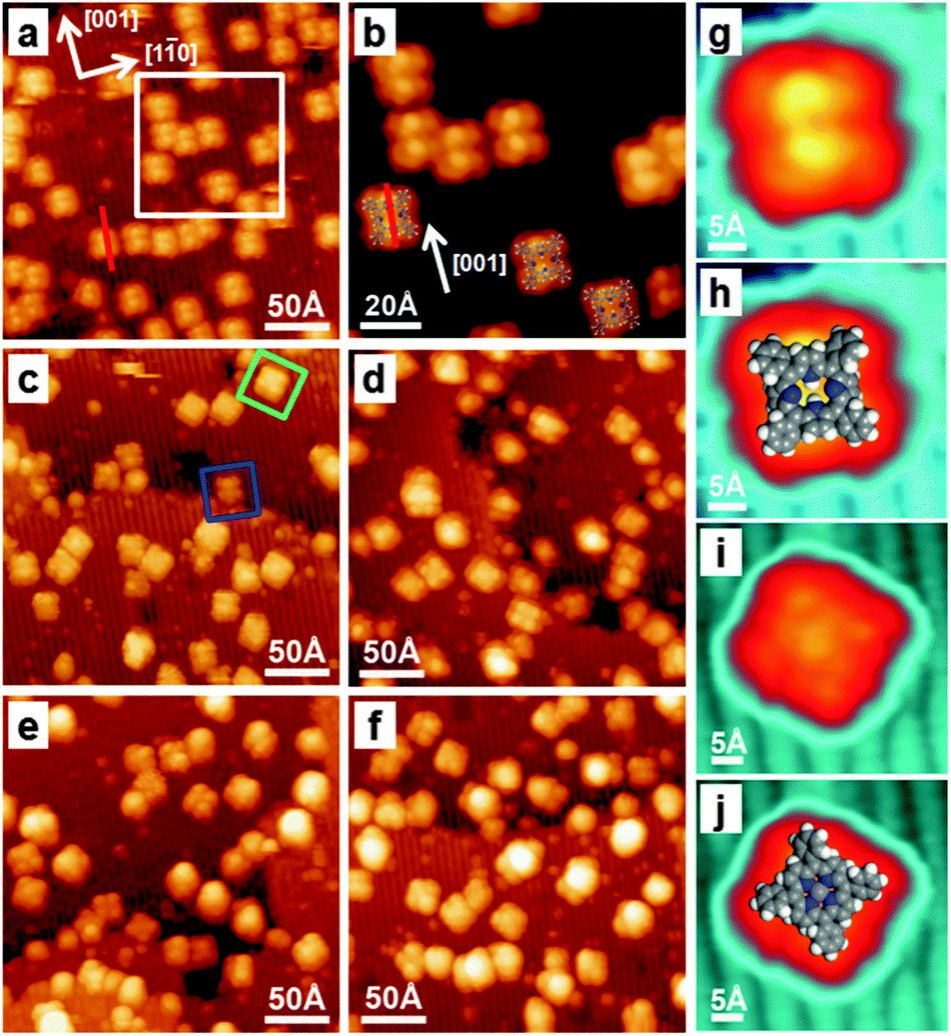
STM images of 2H-TPP and NiTPP molecules on the TiO_2_(110)-(1 × 1) surface. (a) and (b) 2H-TPP molecules imaged before the deposition of Ni atoms. (c–e) STM images obtained after the deposition of Ni atoms with Ni/2H-TPP ratios of 1:1, 2:1 and 3:1, respectively. (f) An image of sample (e) annealed at 550 K. (g,i) High-resolution STM images of 2H-TPP and NiTPP, respectively. (h,j) The same images superimposed with the corresponding molecular models. The figure has been adapted from [53] with permission, copyright 2014 The Royal Society of Chemistry.

Let us start by reviewing research published about metal-free porphyrins. Lovat et al. [52] investigated the adsorption of three differently functionalized metal-free porphyrins, i.e., 2*H*-*tert*-butyltetraphenylporphyrin (2H-TBTPP), 2*H*-tetraphenylporphyrin (2H-TPP) and 2*H*-octaehtylporphyrin (2H-OEP). The different side groups do not introduce a specific affinity for the substrate. The influence of these peripheral terminations is only seen as a change in the height of the central macrocycle of the molecule with respect to the surface. Indeed, the central macrocycle in 2H-TBTPP is ca. 1 Å higher above the substrate than in 2H-OEP; the height for 2H-TPP is found at a point approximately halfway [52]. From the NEXAFS measurements, it is evident that each of the three analysed species adsorbs with the central macrocycle almost parallel to the substrate. Additionally, the authors report the hydrogen uptake from the substrate (hydroxyl groups, hydrogen bulk interstitials) and/or from the residual gas by the molecules in the first layer [52]. Of the above-mentioned porphyrin species, only 2H-TPP has been studied with STM by Lovat et al. [52]. Similar results were reported by Wang et al. [53]. The molecules adsorb with their plane parallel to the substrate when deposited at room temperature, as expected from the results of the NEXAFS measurements. The STM image of a single molecule exhibits two-fold symmetry [52–53]. Molecules are sitting atop the oxygen rows with a common azimuthal orientation in a saddle-shape conformation, which has been identified as the N–N axis parallel to the [001] direction. The most favoured adsorption site is found to be the bridge position between two adjacent oxygen (O_br_) atoms in the row. In this configuration, a 2H-TPP molecule forms two equivalent N–H^…^O_br_ hydrogen bonds. At a low coverage, the molecules do not self-assemble in close-packed islands [52–53]. In addition to studying the adsorption of metal-free porphyrins on the TiO_2_(110)-(1 × 1) surface, Wang et al. [53] investigated the metalation process of 2H-TPP with nickel atoms. The authors showed that it is possible to synthesize in situ NiTPP molecules from vapour-deposited Ni atoms and 2H-TPP molecules. The reaction to form NiTPP from 2H-TPP proceeds at room temperature when the 2H-TPP molecules are deposited first. When the deposition of metal atoms precedes the evaporation of a molecular material, elevated temperatures are needed to trigger the metalation reaction. Interestingly, upon metalation, the conformation and orientation change, and consequently, the STM image of the porphyrin molecule changes as well (Figure 4) [53]. The saddle-shape conformation characteristic of 2H-TPP changes into a flat conformation. Additionally, the NiTPP molecules rotate by 45° with respect to the 2H-TPP species. And finally, the STM image of NiTPP illustrates its four-fold symmetry in contrast to the two-fold symmetry images of 2H-TPP (Figure 4g and Figure 4i).

Only recently a report on metalation of 2H-TPP to NiTPP on TiO_2_(110)-(1 × 2) surfaces has been published [54]. The authors note that post-annealing enhances metalation on the (1 × 2) surface, contrasting to results obtained on a (1 × 1) surface. Metal-free molecules adsorb on the Ti_2_O_3_-added rows along the [001] direction in a tilted configuration and appear as two-lobed features in the STM images. The molecules do not assemble as closed-packed islands and are randomly distributed on the terraces. After the deposition of Ni atoms, metalation reaction takes place. The NiTPP molecules appear in the STM scans as four-lobe adsorbates, and they change their azimuthal orientation by 45° with respect to the 2H-TPP molecules. Interestingly, when the NiTPP molecules were deposited directly on the TiO_2_(110)-(1 × 2) surface the rotation was not observed [54]. Annealing of the sample with 2H-TPP after deposition of Ni atom leads to increase in degree of metalation. NiTPP changes its appearance in the STM images to a more uniform shape resembling the four-fold symmetry of a flat NiTPP molecule. The authors ascribe the difference seen in the STM images of molecules that undergo metalation at room temperature and those annealed at high temperatures to changes in the adsorption sites of the individual molecules induced by the thermal treatment [54]. If the deposition order is reversed, i.e., Ni atoms are deposited first followed by the 2H-TPP molecules, the metalation reactions is also feasible, but the reaction yield is lower. Irrespective of the face on which molecules are deposited, only moderate degrees of metalation are achieved on TiO_2_ surfaces compared to metal surfaces. Wang et al. [54] suggest that this result reflects the influence of the molecule–substrate interactions, i.e., the interactions between the Ni and/or 2H-TPP molecules and the TiO_2_ surfaces.

It is very intriguing to compare the most recent results obtained for zinc porphyrins [55–59]. In contrast to the experiment from Lovat et al. [52] discussed earlier, it is evident that the proper choice of side groups in zinc porphyrins may influence the adsorption behaviour of the molecules. Once the molecules are equipped with carboxyl groups, their adsorption geometry changes from flat lying to upright as the coverage increases – an effect observed on both (110) and (011) surfaces [55–56].

Let us first review the results for zinc protoporphyrin (ZnPP) adsorbed on a rutile TiO_2_(110) surface [55]. To chemically anchoring the molecule to the oxide surface, it is important to consider that carboxylic groups are only on one side of the porphyrin ring. This arrangement allows ZnPP molecules to tilt away from the surface as the amount of deposited material increases. The carboxylic groups deprotonate upon adsorption and form bidentate bonds to the five-fold coordinated titanium atoms on the surface – the molecules are chemically bonded to the surfaces. Similar behaviour has been reported both for small [61–67] and large [68–70] molecules with –COOH groups deposited on TiO_2_. At a low coverage, the ZnPP molecules lay flat on the surface, and the interaction of the porphyrin ring with the surface is a secondary bonding mechanism [55]. An increase in the density of adsorbed molecules leads to changes in the geometry from flat lying to upright. The change is observed for the first monolayer, and the upright geometry is consistently observed for several multilayers until it is no longer present for thick films. When the coverage includes several layers, deprotonation is observed only for molecules in the layer closest to the substrate. The interaction of first layer molecules with the substrate surface seems to be quite strong, despite the reorientation of ZnPPs at high-coverage levels. Indeed, Rienzo et al. [55] report that Zn atoms are pulled from the molecules in the first layer. It is expected that protons released during the deprotonation of –COOH groups displace the metal atoms, and the molecules form the metal-free porphyrin. This process has serious implications because it changes the inherent chemical and physical functionalities of the metal porphyrins in the layer.

**Figure 5:**
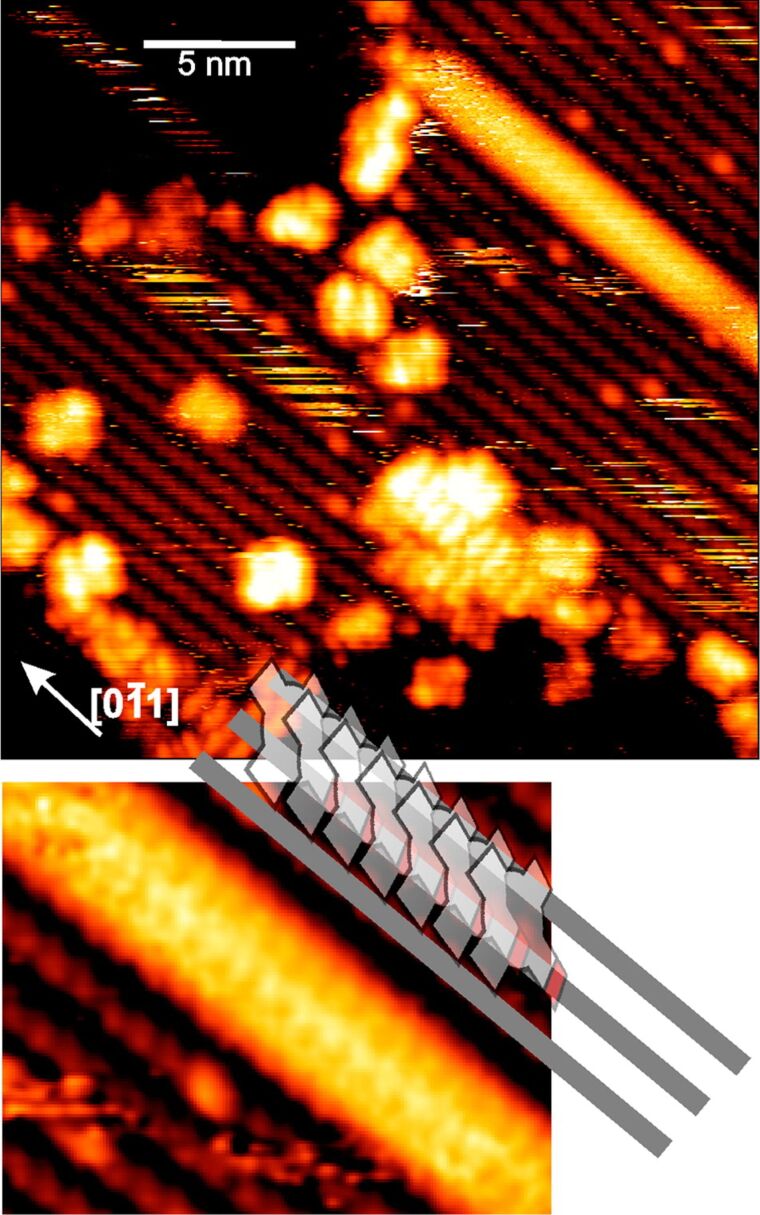
A linear π–π-stacked structure formed by COOH-ZnTPP molecules on a TiO_2_(011)-(2 × 1) surface – see in the upper right corner of the main image. The lower panel is a detailed view of the 1D line together with a scheme of the proposed molecular organization in the assembly. The figure has been adapted with permission from [56], copyright 2015 American Chemical Society.

The influence of carboxyl groups on the behaviour of the porphyrin molecules has also been studied with STM [56–57]. Olszowski et al. [56] compared the adsorption of meso-tetraphenylporphyrinzinc(II) (ZnTPP) with 5-(4-carboxyphenyl)-10,15,20-triphenylporphyrinzinc(II) (COOH-ZnTPP) on the (011) surface of TiO_2_. At a low coverage, ZnTPP molecules adsorb in a flat-lying conformation and are very mobile on the surface, with their motion limited to surface reconstruction rows, i.e., along the [0−11] direction. There is a fraction of molecules found to be in stable positions; however, they are so weakly bound that even mild scanning conditions can easily perturb them. As the coverage density increases, the molecules become sterically trapped, and a layer of flat-lying molecules is formed. Yet, the layer does not show long-range order: The molecules are forming short lines along the surface rows or a chessboard pattern, but overall the layer is rather quasi ordered. For the closed monolayer, the molecules remain flat lying. The situation changes considerably once a single carboxyphenyl group is introduced instead of one phenyl group. At a low coverage, the COOH-ZnTPPs are also adsorbed in a flat-lying geometry and exhibit pronounced mobility. However, a considerable number of molecules are found immobilized at surface defects or domain boundaries, and these molecules can hardly be manipulated by the tip [56]. Already, at a low coverage, some of the molecules form 1D π–π-stacked structures along the [0−11] direction (Figure 5). In this structure, COOH-ZnTPP molecules lift their porphyrin ring up leaving –COOH oriented toward the substrate. As the coverage increases, the number of 1D stacks grows until a monolayer of upright-oriented molecules is formed. The authors suggested that the molecular planes are not perpendicular to the surface plane but are inclined at some angle [56]. However, from the STM measurements alone, it is hard to establish that angle. Finally, Olszowski et al. [56] stated that the –COOH groups remain intact upon adsorption on the TiO_2_(011) surface.

Only recently, Zajac et al. [57] have compared the adsorption of ZnTPP and COOH-ZnTPP on the (110) surface of TiO_2_. Both molecules adopt a planar configuration. At a submonolayer coverage, COOH-ZnTPP molecules can be imaged with STM at room temperature. On the contrary, the ZnTPP molecules are easily disturbed by the scanning tip, which hinders high-quality microscopic analysis. As the coverage density increases, both species form stable islands with flat-lying molecules and rhomboid unit cells. For even higher coverage, the authors did not observe changes in the adsorption geometry for molecules equipped with –COOH groups. That result is puzzling in view of the earlier discussed reports [55–56]. It seems that Zajac et al. [57] did not achieve the coverage density at which the reorientation is feasible.

For completeness, we recall here the latest results obtained for ZnTPP molecules deposited onto the TiO_2_(110)-(1 × 1) surface [58]. The authors studied the formation, structure and energy level alignment of the first monolayer. To increase the chances of forming an ordered layer, Rangan et al. deposited large amount of molecules onto a substrate kept at room temperature and then annealed the sample at 150–200 °C [58]. As a result, they obtained terraces nearly completely covered with molecules arranged in a rectangular lattice. Within the lattice, in general, individual molecules lay flat with their central Zn atom located above an oxygen row and opposite pyrroles of the macrocycle oriented along that row. The molecule is slightly distorted upon adsorption, i.e., the phenyl rings of the side groups are rotated to allow for weak hydrogen bonds to form with the surface oxygen atoms. Additionally, the authors note that on the surface, two different ordered domains are encountered. These domains are symmetrical with respect to a (1−10) mirror plane. Rangan et al. also employed spectroscopic techniques to study the energy levels alignment and compared their results with theoretical calculations. They concluded that the adjustment of the energy levels is highly sensitive to the coverage density of the molecular material. Additionally, mutual molecular interactions stabilize a densely packed monolayer with a reduced molecule–surface distance. Consequently, charge transfer from the molecule to the oxide takes place, and an interface dipole is formed.

To further understand the energy level alignment between a semiconducting substrate and an organic adsorbate, Lackinger, Janson and Ho [59] studied interactions between zinc(II) etioporphyrin (ZnEP) and oxygen vacancies, which are point defects commonly found in TiO_2_(110) surfaces. The energy level alignment is of crucial importance for DSSC applications of titania. The authors took special care to prepare a sample with unsaturated oxygen vacancies [59] because it is known that they can be easily passivated even at very low partial pressures of water in a well baked ultrahigh vacuum system [71]. For further clarity, they performed measurements at low temperatures (ca. 11 K), i.e., conditions that critically reduce the molecular mobility. At low temperatures, ZnEP molecules adsorbed on the TiO_2_(110) surface are found in two conformations – a planar, seen as a four-lobed feature, and a non-planar, seen as a two-lobed feature. Additionally, these conformations differ in their d*I*/d*V* spectra, i.e., the non-planar shows the LUMO resonance at positive sample bias, and the planar shows the HOMO resonance at negative sample bias. The authors related the non-planar conformation to the ability to stabilizing different geometries with rotatable side groups, i.e., ethyl and methyl side groups [59]. Low temperature measurements are suitable for experiments with controlled tip manipulation. Indeed, Lackinger, Janson and Ho [59] were able to move ZnEP molecules in both conformations with the STM tip in order to change their distance from neighbouring oxygen vacancies. These manipulation events were accompanied with scanning tunnelling spectroscopy measurements revealing the significant influence of oxygen vacancies on the position of the electronic states of molecules in the planar conformation. If molecules in the planar configuration are positioned on an oxygen vacancy, the HOMO resonance shifts outward from the Fermi level by about −0.4 V with respect to that of the molecule sitting on non-defected area [59]. The HOMO−1 state of the same molecule is hardly affected by the presence of the defect. As soon as molecules in the non-planar configuration are moved onto an oxygen vacancy, the LUMO state is only slightly shifted with respect to its position for molecules located away from oxygen vacancies. The authors also discussed the possible origins of the observed energy shift, however, without a clear conclusion. Regardless of the mechanism responsible for the observed effect, the results presented by Lackinger, Janson and Ho [59] are critically important to DSSC applications. In the DSSCs the proper level alignment of the dye and the substrate is fundamental for their efficient operation. The authors showed that inherent point defects present on the TiO_2_(110) surface, i.e., oxygen vacancies, can significantly influence the position of the electronic states of the adsorbates. Once again, it has been demonstrated that the conformation adopted by the molecule has an impact on the electronic level alignment.

The level shifts observed by Lackinger, Janson and Ho [59] may be related to the presence of delocalized excess electrons in the subsurface layers [36–41], discussed earlier in the context of experiments on PTCDI molecules (see section “PTCDA molecules”). As Lanzilotto et al. [35] have shown, the interaction range for these electrons is limited to a single unit cell. Consequently, when considering a molecular layer only single molecules are influenced by the subsurface delocalized charges. Thus, in view of these results, the next step should be aimed on monitoring the influence of the oxygen vacancies density on the averaged level alignment between a semiconducting substrate and a full organic layer.

Another method used to understand the molecule-substrate interactions is Kelvin probe force microscopy (KPFM). Jöhr et al. used non-contact atomic force microscopy and KPMF to study the adsorption and interaction of copper(II) meso-tetra(4-carboxyphenyl)porphyrin (Cu-TCPP) on a TiO_2_(110) surface in a submonolayer coverage regime at room temperature [60]. First, the authors identified two dominant adsorption geometries. In both of them, the molecules lay flat, with their central macrocycle parallel to the substrate surface. These two geometries differ in their azimuthal angles. In one of them, the molecule is oriented in such a way that the central copper atom is located over the titanium row, and two opposite carboxyphenyl groups are aligned along the [001] direction forming two covalent bonds between the carboxyl groups and the surface five-fold coordinated titanium atoms in a monodentate fashion; the other two carboxyphenyl groups are aligned along the [1−10] direction. In the second geometry, Cu-TCPPs are rotated in-plane by 45°, and their central copper atoms are located over bridging oxygen atoms; the molecule does not form covalent bonds with the substrate. Further, the authors corroborated their results with theoretical calculations and concluded that the carboxyl groups stay intact in both geometries, i.e., there is no deprotonation [60]. From KPFM and the calculations, Jöhr et al. concluded that a charge is transferred to the substrate upon adsorption and a dipole moment that points away from the surface is formed, regardless of the adsorption geometry. The presence of this type of dipole moment in the first molecular layer of dye could negatively influence the DSSC performance because it may decrease the electron injection rate to the conduction band of the titania substrate [60].

### Examples of heteromolecular systems

Lastly, we want to examine two interesting heteromolecular systems comprising molecules that are aligned with the given review. The first system comprises FePc molecules deposited on a monolayer of bipyridine (BiPy) used as a buffer layer [48], and the second is composed of CuPc molecules deposited on a wetting layer made of ZnTPP molecules [49].

The study of the FePc/BiPy/TiO_2_(110) system [48] is an extension of an experiment of the adsorption characteristics of FePc molecules on the TiO_2_(110) surface [45]. From the latter research, it was concluded that FePcs adsorb on the (110) face of rutile titania in a flat geometry to form a layer of molecules that strongly interact with substrate (i.e., molecules are oxidized, and the molecular orbitals are severely influenced) [45]. In the second layer, molecules form islands, and their electronic structures are less disturbed. The introduction of a BiPy layer aimed to decouple the first layer of FePc molecules from the oxide substrate [48]. Indeed, the BiPy molecules formed a buffer layer of upright molecules that, by spatial separation of FePc from the TiO_2_(110) surface, allows for its electronic decoupling. As soon as iron phthalocyanine molecules are deposited on the buffer layer, they adsorb and form molecular chains in a slightly tilted geometry along the [001] direction. More importantly, their electronic structure is neither altered by the interaction with substrate nor by interaction with the underlying molecular layer. Finally, the authors showed that the level alignment of the FePc/BiPy/TiO_2_(110) system is compatible with the characteristics required of DSSC applications [48]. In particular, the LUMO of FePc is above the substrate conduction band minimum, and the LUMO of BiPy is located between these two states. This configuration should allow electron transfer from the exited state of the FePc through the BiPy layer into the conduction band of the substrate. Additionally, the HOMO of BiPy is located significantly below the HOMO of FePc, and therefore, it is expected that substrate–dye charge recombination would be hampered.

The last example involves the formation of a CuPc molecule overlayer on a wetting layer of ZnTPP molecules deposited on TiO_2_(011) [49]. We have already discussed the results of experiments on both CuPc and ZnTPP molecules deposited onto rutile surfaces [33,43,56]. In the former case, the CuPc molecules formed a wetting layer of flat-lying molecules on which ordered islands formed [33,43]. Furthermore, it was possible to reorient all of the molecules in the second layer to an upright geometry, a conformation that may critically influence the molecule–substrate interactions [43]. Unfortunately, cryogenic temperatures were necessary for probing the layered structure with STM [33,43]. One of the prerequisites of DSSCs, however, is the ability to function at least at room temperature. The introduction of ZnTPP molecules as a buffer layer changes the stability of the CuPc overlayers dramatically, finally allowing high-resolution room temperature STM measurements [49]. Let us recall that ZnTPPs form a quasi-ordered layer of flat-lying molecules [56]. First, the deposition of a submonolayer of CuPc molecules at room temperature onto a ZnTPP wetting layer resulted in unordered growth of molecular clusters. However, once the sample was annealed at 150 °C, the unordered structures transmuted into ordered islands of upright standing molecules. The authors observed that the subsequent deposition of molecular material followed by thermal annealing at the same temperature eventually led to the formation of a well-ordered layer of upright standing CuPc molecules that covered the whole surface. The limiting factor appeared to be the lateral dimensions of the underlying substrate terraces [49]. Further deposition resulted in the formation of a second layer of CuPcs that exhibited an even higher degree of order than the first layer. A comparison of the experiments reported by Godlewski et al. [33,43] and Zając et al. [49] illustrates that heteromolecular overlayers are likely more stable than their homomolecular counterparts, and thus, they are worth considering in the design of the organic photovoltaic devices.

## Conclusion

From a review of past research, the first layers of PTCDA, phthalocyanine and porphyrin molecules share similar single-molecule adsorption geometries. The molecules tend to grow with their molecular plane nearly parallel to the substrate, as long as they do not possess specific anchor groups, e.g., –COOH. Each of the discussed molecular species possesses the highly delocalized π-orbitals extending in the direction perpendicular to the molecular plane. In the flat-lying arrangement, these orbitals play a pivotal role in the molecule–substrate interactions, thus, mediating the charge injection into the substrate. However, once carboxylic groups are judiciously introduced, the molecules tend to realign in an upright position as the coverage density increases. In such a case, molecules often form π-stacked structures, thus, the mutual π–π interactions between molecules moderate the intermolecular charge transport within the organic structure. The charge injection and charge transport both are very important when considering potential applications in photovoltaics, electronics and other fields.

Additionally, inherent surface defects, such as oxygen vacancies on the TiO_2_(110) surface, cannot be ignored because they affect the level alignment of the molecule–oxide interface. Furthermore, delocalized charges formed by excess electrons redistributed among multiple Ti lattice sites in the subsurface layers have been shown to lead to the reduction of some molecules in the first layer. The first layer of adsorbate is intrinsically prone to strongly interact with the substrate. Thus, the level alignment at the interface should be considered as an emerging property of composite system, i.e., organic layer/TiO_2_. A possible method for tailoring the level alignment in dye-sensitized devices is to introduce a buffer layer between the oxide electrode and the layer of dye molecules. The careful selection of buffer layer molecules may lead to systems with unaffected charge transport from the excited states of dye molecules to the oxide electrode, with significantly diminished dye–substrate recombination, and increased stability and quality of the active top-most layer.

Understanding of the molecule–substrate interactions is one of the key elements of successful design of organic DSSCs. In this regard, in addition to study the adsorption of organic dyes on the surfaces of wide-band-gap materials with the use of scanning tunnelling microscopy or atomic force microscopies, it is indispensable to use other techniques such as Kelvin probe force microscopy, which allows measuring the local work function with high resolution. Such a measurement may shed some light on fundamental processes taking place in an organic DSSC upon photon absorption.

Quite often, in prototypical and commercial applications, TiO_2_ is used in powder form (the most common is Degussa P25). In such a nanopowder, both rutile and anatase forms are present. There is a huge discrepancy in the state of knowledge on different crystal forms of titanium dioxide. With respect to real applications, it is imperative to investigate the adsorption of organic dyes on different faces of anatase titania.

Scanning probe microscopic studies provide excellent insight into the local environment of a single dye molecule, thereby illuminating the fundamental processes governing dye-sensitized photovoltaic devices. In combination with common spectroscopic methods, we gain a better understanding of the mechanisms taking place in DSSCs and can take advantage of this knowledge to design improved devices. In the end, it is all about achieving the highest possible device efficiency.
